# Targeting noncanonical nuclear factor kappa B signalling in *CYLD* cutaneous syndrome by selective inhibition of IκB kinase alpha

**DOI:** 10.1093/bjd/ljag044

**Published:** 2026-03-04

**Authors:** Kirsty Hodgson, Joseph Inns, Gary Reynolds, Emily Stephenson, Andrew Paul, Naomi Sinclair, Giacomo Berretta, Christopher Lawson, Andrew Michael Frey, Iglika Ivanova, Eva Adam, Christopher J Lord, Simon Cockell, Jonathan Coxhead, Nikoletta Nagy, David Adams, Marta Szell, Matthias Trost, Muzlifah Haniffa, Simon P Mackay, Neil Perkins, Neil Rajan

**Affiliations:** Translational and Clinical Research Institute, Newcastle University, Newcastle upon Tyne, UK; Translational and Clinical Research Institute, Newcastle University, Newcastle upon Tyne, UK; Biosciences Institute, Newcastle University, Newcastle upon Tyne, UK; Centre for Immunology and Inflammatory Diseases, Massachusetts General Hospital, Boston, MA, USA; Biosciences Institute, Newcastle University, Newcastle upon Tyne, UK; Strathclyde Institute of Pharmacy and Biomedical Sciences, University of Strathclyde, Glasgow, UK; Translational and Clinical Research Institute, Newcastle University, Newcastle upon Tyne, UK; Strathclyde Institute of Pharmacy and Biomedical Sciences, University of Strathclyde, Glasgow, UK; Strathclyde Institute of Pharmacy and Biomedical Sciences, University of Strathclyde, Glasgow, UK; Biosciences Institute, Newcastle University, Newcastle upon Tyne, UK; Biosciences Institute, Newcastle University, Newcastle upon Tyne, UK; Department of Medical Genetics, University of Szeged, Szeged, Hungary; HUN-REN-SZTE Functional Clinical Genetics Research Group, Szeged, Hungary; The CRUK Gene Function Laboratory and Breast Cancer Now Toby Robins Research Centre, The Institute of Cancer Research, London, UK; Biosciences Institute, Newcastle University, Newcastle upon Tyne, UK; Biosciences Institute, Newcastle University, Newcastle upon Tyne, UK; Department of Medical Genetics, University of Szeged, Szeged, Hungary; HUN-REN-SZTE Functional Clinical Genetics Research Group, Szeged, Hungary; Wellcome Sanger Institute, Wellcome Genome Campus, Cambridge, UK; Department of Medical Genetics, University of Szeged, Szeged, Hungary; HUN-REN-SZTE Functional Clinical Genetics Research Group, Szeged, Hungary; Biosciences Institute, Newcastle University, Newcastle upon Tyne, UK; Biosciences Institute, Newcastle University, Newcastle upon Tyne, UK; Wellcome Sanger Institute, Wellcome Genome Campus, Cambridge, UK; Department of Dermatology and NIHR Newcastle Biomedical Research Centre, Newcastle Hospitals NHS Foundation Trust, Newcastle upon Tyne, UK; Strathclyde Institute of Pharmacy and Biomedical Sciences, University of Strathclyde, Glasgow, UK; Biosciences Institute, Newcastle University, Newcastle upon Tyne, UK; Translational and Clinical Research Institute, Newcastle University, Newcastle upon Tyne, UK; Department of Dermatology and NIHR Newcastle Biomedical Research Centre, Newcastle Hospitals NHS Foundation Trust, Newcastle upon Tyne, UK

## Abstract

**Background:**

*CYLD* cutaneous syndrome (CCS) skin tumours develop from puberty onwards, can number in the hundreds and progressively grow over time. Patients with CCS lack medical therapies and require repeated surgery to control tumour burden. *CYLD* loss of heterozygosity drives tumour growth, and CCS tumours have previously been shown to demonstrate increased canonical nuclear factor kappa B (NF-κB) and Wnt signalling.

**Objectives:**

To investigate NF-κB signalling in CCS tumours and CCS tumour keratinocytes, with the aim of identifying druggable targets.

**Methods:**

We used complementary bulk transcriptomics and proteomics in patient-derived CCS tumour cell fractions, as well as single-cell RNA sequencing of CCS tumour cells to investigate aberrant NF-κB signalling. We developed a patient-derived CCS tumour spheroid culture model to determine the utility of targeting aberrant NF-κB cell signalling.

**Results:**

We demonstrate evidence of non-canonical NF-κB signalling in CCS tumour keratinocytes, with increased p100 to p52 processing and RelB protein expression compared with normal skin. We identify IκB kinase alpha (IKKα) as a candidate target in the noncanonical NF-κB signalling pathway. A novel, highly selective IKKα inhibitor (SU1644) used in patient-derived CCS tumour spheroid cultures demonstrated that IKKα inhibition reduced tumour spheroid viability.

**Conclusions:**

These data provide the preclinical rationale for the assessment of topical IKKα inhibitors as a novel preventive treatment for CCS.

## Graphical Abstract

**Graphical Abstract:**
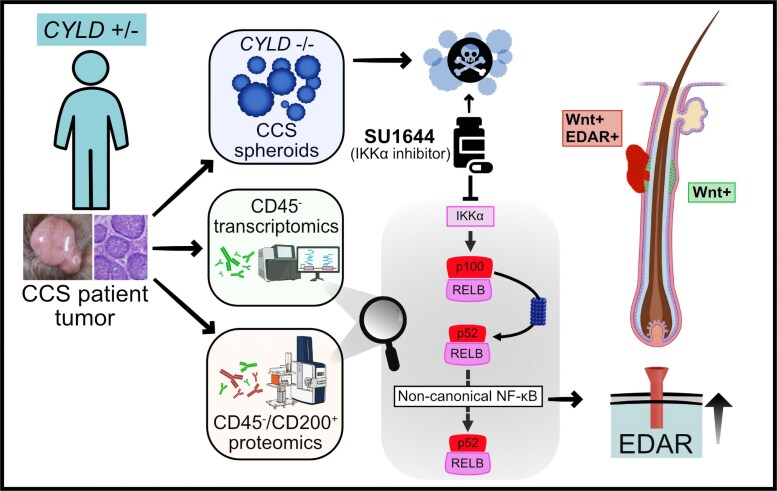

## Teaser

Topical IKKα inhibition emerges as a potential therapy for *CYLD* cutaneous syndrome by targeting noncanonical NF-κB signalling.

What is already known about this topic?

- *CYLD* cutaneous syndrome (CCS) is an inherited condition caused by pathogenic variants in *CYLD*, leading to uncontrolled nuclear factor kappa B (NF-κB) signalling and tumour growth.
- Patients develop multiple benign skin tumours (cylindromas and spiradenomas) on the head and torso.
- There are no preventive treatments, and repeated surgeries significantly impact patients’ quality of life.

What does this study add?

- While CCS tumours were previously known to exhibit increased canonical NF-κB and Wnt signalling, we provide evidence of dysregulated noncanonical NF-κB signalling in CCS tumour keratinocytes, marked by increased p100 to p52 processing and RelB expression.
- Through transcriptomic and proteomic analyses, we identify IκB kinase alpha (IKKα) as a key mediator of noncanonical NF-κB signalling in CCS tumour keratinocytes.
- Targeting IKKα with a selective inhibitor (SU1644) in patient-derived spheroid models reduced tumour spheroid viability, suggesting its potential as a druggable target.

What is the translational message?

- By demonstrating that IKKα inhibition can suppress CCS tumour growth *in vitro*, we lay the foundation for the development of topical IKKα inhibitors as a preventive and noninvasive treatment for CCS, addressing a critical unmet medical need in dermatology.

*CYLD* cutaneous syndrome (CCS) is a dominantly inherited skin tumour syndrome driven primarily by pathogenic variants (PVs) in *CYLD*. Most patients with CCS carry germline heterozygous truncating *CYLD* PVs involving the catalytic domain. Subsequent loss of heterozygosity and CYLD inactivation leads to hair follicle tumours (cylindromas and spiradenomas) with high CD200 expression.^1,4^ CCS tumours appear on the head and torso,^5^ enlarging from puberty onwards.^6^ Benign CCS skin tumours can transform, invading the skull, metastasizing to the lung and other sites,^7^ resulting in death. Patients with CCS undergo lifelong surgical procedures that may culminate in scalp removal. Patients have stated that the significant impact of CCS on their quality of life is poorly understood by the medical profession.^8^ There are no pre-emptive therapies for *CYLD* PV carriers, and CCS represents an unmet medical need.^9^

*CYLD* is a tumour suppressor, epigenetically repressed or mutated in leukaemia, lymphoma, myeloma, neuroblastoma and liver cancer.^10–14^ Somatic truncating CYLD mutations are documented in head-and-neck squamous cell carcinoma and anal carcinoma,^15,16^ underscoring the relevance of studying the signalling consequences of *CYLD* loss-of-function mutations.

CYLD is a deubiquitinase that hydrolyses Lys63- and Met1-linked polyubiquitin chains,^17–19^ and is a negative regulator of canonical nuclear factor kappa B (NF-κB) signalling,^20,21^ with targets spanning the pathway [tumour necrosis factor (TNF) receptor 1, T-cell receptor, B-cell receptor, Toll-like receptor 2 and IκB kinase (IKK)].^22^ CYLD loss promotes RelA/p50 dimerization and transcription of canonical target genes.^20,21,23–26^ CYLD also negatively regulates noncanonical NF-κB signalling, where NF-κB-inducing kinase and IKKα dimerization promotes p100 to p52 processing and p52/RelB-mediated transcription of target genes.^27^ In murine keratinocytes, CYLD prevents nuclear translocation of BCL-3-p50/BCL-3–p52 complexes via BCL-3 deubiquitination.^28^ Increased RelB and p100 levels have been found in B cells from Cyld knockout and knock-in mouse models expressing a short splice variant of murine *Cyld* lacking exons 7 and 8.^26,29^ Additionally, B cells from mice expressing catalytically inactive Cyld exhibited increased nuclear p52 compared with control cells,^30^ suggesting a possible undescribed role for noncanonical NF-κB signalling in CCS tumour keratinocytes.^31,32^

In CCS tumours, CYLD negatively regulates canonical NF-κB, Wnt/β-catenin, tropomyosin receptor kinase and transforming growth factor-β signalling pathways.^2,33,35^ Furthermore, sporadic spiradenoma and spiradenocarcinoma skin tumours without *CYLD* PVs are frequently driven by a somatic *ALPK1* hotspot mutation (p.V1092A), which also enhances NF-κB activation, underscoring the importance of NF-κB signalling in CCS-related tumour pathogenesis.^36^ A comprehensive understanding of the impact of truncating CYLD PVs on NF-κB signalling in CCS tumours has been hampered by the lack of mouse models that faithfully recapitulate the CCS skin tumour phenotype.^28,37^ To overcome this, we used patient tumours driven by natural mutagenic events affecting *CYLD*, offering a cellular signalling context relevant to the discovery of therapeutic targets in CCS.

Using transcriptomic and proteomic profiling of CCS tumours, we demonstrate enriched noncanonical NF-κB signalling in CCS tumour keratinocytes. Ectodysplasin A receptor (EDAR) was the most differentially expressed noncanonical NF-κB signalling transcript, an activator of canonical NF-κB signalling in CCS tumour keratinocytes.^38^ To investigate this, we developed a novel patient-derived CCS tumour keratinocyte-enriched spheroid model. We targeted noncanonical signalling in CCS spheroids by inhibiting IKKα with SU1644, a novel, highly selective IKKα inhibitor, which reduced *EDAR* expression and CCS spheroid viability at low micromolar concentrations. These data highlight IKKα as a candidate druggable target in CCS and support future studies of topical, highly selective IKKα inhibitors in patients with CCS.

## Materials and methods

### Skin samples

Skin samples were obtained from patients with CCS (Table S1; see Supporting Information).

### Spheroid culture

Primary cells were cultured in media supplemented with 1.5 mmol L^–1^ CaCl_2_/10 μmol L^–1^ ROCKi (5 × 10^5^–2 × 10^6^ cells per well) in Aggrewell400™ six-well plates (STEMCELL Technologies, Cambridge, UK) with an initial centrifugation at 100 ***g*** for 3 min. Media was changed after 48 h by aspirating and replacing with media containing 1.5 mmol L^–1^ CaCl_2_.

### RNA sequencing library preparation

RNA was extracted from samples sorted by fluorescence-activated cell sorting (FACS) with an RNeasy Micro Kit (QIAGEN, Santa Clarita, CA, USA) and bulk tissue with an Allprep DNA/RNA/miRNA Universal Kit (QIAGEN) following the manufacturer’s protocols. TapeStation (Agilent, Santa Clara, CA, USA) RNA quality control and stranded preparation with the NEBNext® Low Input RNA Library Prep Kit for Illumina® (New England Biolabs, Hitchin, UK) was performed. Libraries were single-end sequenced using a NextSeq 550 system (Illumina, San Diego, CA, USA). Single-cell RNA sequencing (scRNAseq) library generation was performed with a Chromium Single Cell 5′ GEM Library (10X Genomics, Pleasanton, CA, USA) generation kit, according to manufacturer protocols. Libraries were pair-end sequenced using an S1 flow cell and Illumina Novaseq system.

### Bioinformatic analysis

Bulk RNA sequencing data quality was assessed with FastQC (https://github.com/s-andrews/FastQC) and reads were aligned using the splice-aware aligner program STAR.^39^ Aligned sequencing reads were mapped to genomic features and counted using the Python package ‘HTSeq’.^40^ Count data were filtered to remove genes with a total read count < 15, normalized by the trimmed mean of M values method in the Bioconductor package ‘edgeR’,^41^ and then transformed by ‘voom’ using ‘limma’.^42,43^ Differential gene expression analysis was performed using DeSeq2.^44^ Functional enrichment analysis of differentially expressed genes (DEGs) was performed with the webserver g:Profiler.^45^ scRNAseq base call files (BCLs) were processed using Cellranger 3.0.1 and aligned to Cellranger reference GRCh38 3.0.0. Cells with < 200 genes or > 10% mitochondrial transcripts were filtered. Batch correction by sequencing lane was performed using Harmony (harmonypy v. 0.0.9; https://pypi.org/project/harmonypy/0.0.9/). Gene scoring per cell of the 49 DEGs with p52/RelB transcription factor binding sites (TFBS) was performed using the tl.score.genes Scanpy module (https://scanpy.readthedocs.io/en/stable/- https://github.com/scverse/scanpy).

### Statistical analysis

Additional data analysis, including statistical tests and visualizations, were performed in the R computing environment (v. 4.3.0; R Foundation for Statistical Computing, Vienna, Austria), using the ‘limma’, ‘tidyverse’ and ‘ggplot2’ packages.^43,46,47^ Reverse transcription quantitative polymerase chain reaction analysis was performed in Prism (GraphPad, La Jolla, CA, USA). Statistical tests used and *P*-values are noted in the figure legends. Further materials and methods are detailed in Appendix S1 (see Supporting Information).

## Results

### Noncanonical NF-κB signalling in CCS tumours drives *EDAR* overexpression

**Figure 1:**
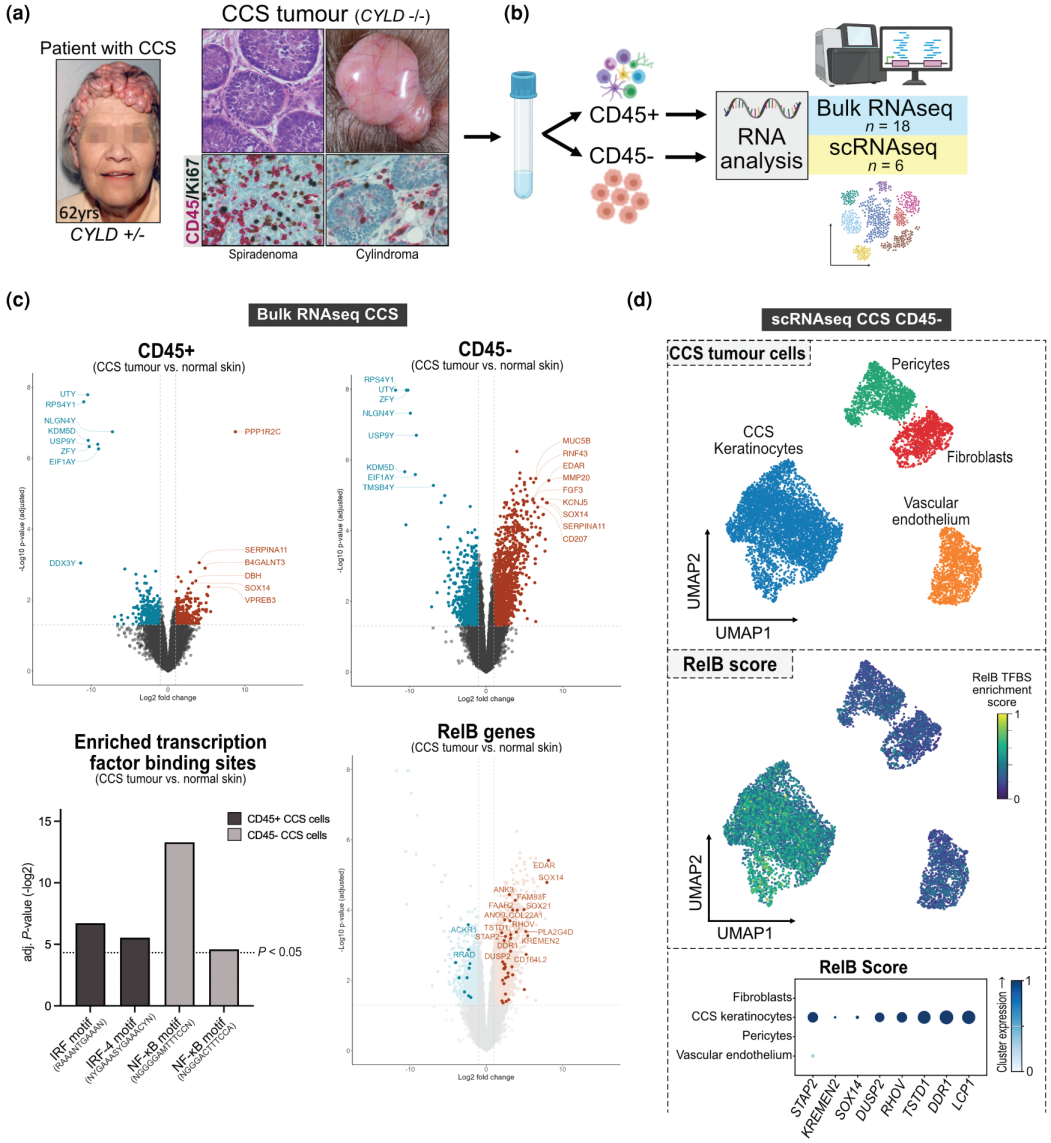
Expression of *EDAR* and *EDA*/*EDAR* target genes in CD45^–^ enriched CYLD cutaneous syndrome (CCS) transcriptomics. (a) Left panel: patients with CCS develop tumours predominantly across the scalp and face from puberty onwards, with an autosomal dominant pattern of inheritance. Right upper panels: haematoxylin and eosin-stained section of a cylindroma and a clinical image of a cylindroma scalp tumour. Right lower panels: immunohistochemical expression of CD45 (pink) and Ki67 (brown) cells shown in a representative spiradenoma and cylindroma. Microscopy images captured at × 40 magnification. (b) Flow cytometry of enzyme-disassociated CCS tumour and control skin suspensions was used to generate CD45^+^ and CD45^–^ single-cell populations that were used to create bulk and single-cell libraries for RNA sequencing (RNAseq). (c) Upper panels: differential gene expression between CCS tumours and healthy skin was analysed in CD45^+^ and CD45^–^ fractions from bulk RNAseq data and visualized as volcano plots. Lower left panel: the 500 genes with the highest increase in expression in CD45^+^ and CD45^–^ CCS tumour fractions were input into g:Profiler for functional enrichment analysis, where statistically significant transcription factor motif matches were found (Benjamini–Hochberg corrected *P*-values). Lower right panel: RelB target genes overlaid on CD45^–^ differentially expressed gene (DEG) volcano plot. (d) Upper panel: uniform manifold approximation and projection (UMAP) plot of unsupervised clustering of CD45^–^ cells from CCS tumours (*n* = 3) according to similarity of transcriptome resulting in four clusters. Middle panel: gene scoring per cell of the 49 DEGs with the p52/RelB transcription factor binding site (TFBS) was performed and indicated as a RelB score overlaid onto a UMAP plot. Lower panel: dot plot displaying mean gene expression for each cluster (colour) and percentage of cells expressing the marker (RelB score markers) within a cluster (dot size). IRF, interferon regulatory factor; NF-κB, nuclear factor kappa B; scRNAseq, single-cell RNA sequencing.

CCS skin tumours were first investigated to determine CD45 expression, demonstrating varying levels of CD45^+^ cell infiltration in the cylindroma and spiradenoma tumour microenvironment (Figure 1a). As CD45^+^ immune cells include lineages that express NF-κB target genes,^48^ we depleted CCS tumour cells of infiltrating CD45^+^ cells by CD45-labelling enzymatically digested CCS skin tumour cell suspensions followed by FACS, an established approach to studying CD45^–^ tumour cells in cancer.^49^ RNA sequencing analysis was performed on CD45^+^ (*n* = 9) and CD45^–^ (*n* = 9) fractions, with additional samples sequenced for quality control purposes [Figure 1b; Table S1, Figure S1 (see Supporting Information)].

Analysis of RNA transcripts identified 1049 and 3427 DEGs in CD45^+^ and CD45^–^ CCS tumour cells, respectively, compared with their healthy skin CD45^+^ and CD45^–^ counterparts [log2 fold change (FC) > 1, adjusted *P*-value (*P*_adj_) < 0.05; Figure 1c]. CCS tumour CD45^–^ cells showed increased expression of *KRT8*, *KRT17* and hair follicle cytokeratin *KRT74* [*P*_adj_ < 0.05; Figure S2a (see Supporting Information)], confirming that CCS CD45^–^ fractions were enriched in CCS tumour keratinocytes.^7^ Gene Ontology analysis revealed that CCS CD45^–^ cells were enriched in epidermis development genes, while CCS CD45^+^ cells were enriched in cell surface receptor pathway genes (Figure S2b). Functional enrichment analysis of DEGs in CCS CD45^+^ and CD45^–^ cell fractions identified upregulated genes with NF-κB TFBS in the TRANSFAC PWM library and MATCH.^50^ The top match was the p52/RelB heterodimer motif (NGGGGAMTTTCCNN) in the CD45^–^ cell fraction, indicative of upstream noncanonical NF-κB signalling (Figure 1c). In total, 798 putative genes with at least 1 p52/RelB TFBS (motif GGGGNTTTCC) ± 1 kb from the transcriptional start site (TSS) were identified. Next, we selected genes with a p52/RelB TFBS differentially expressed in CD45^–^ CCS tumour cells [FC > 2, *P*_adj_ < 0.05 (Benjamini–Hochberg)]. Of 1767 DEGs, 49 had a p52/RelB motif ± 1 kb from the TSS. *EDAR* was the most differentially expressed [8.2 log2FC; Figure S3, Table S2 (see Supporting Information)]. EDAR drives canonical NF-κB signalling in hair follicle development and is overexpressed in Wnt/β-catenin-mediated breast cancers,^51^ leading us to assess genes it modulates in CCS tumours. We analysed differential expression of a literature-curated set of EDAR signalling pathway genes in the bulk CD45^–^ cell data.^52–55^ Consistent with active EDAR signalling, 16 candidate EDAR-regulated genes were differentially expressed in CCS tumour samples vs. healthy control samples, including *KREMEN2*, *WNT10B* and *TNFRSF18* (Figure S2c).

We generated a ‘RelB score’ from the p52/RelB TFBS genes differentially expressed in CD45^–^ CCS tumour cells, which we explored at a single-cell level to confirm whether expression of these genes originated from CCS tumour keratinocytes. We characterized the cell populations in the CD45^–^ CCS samples (*n* = 3 samples; K = 7349 cells), which included CCS tumour keratinocytes (*KRT14*, *KRT17*), fibroblasts (*LUM*, *CFD*), pericytes (*RGS5*, *GJA4*) and CCS vascular endothelial cells (*PECAM1*, *ADGRL4*) (Figure 1d). CCS tumour keratinocytes had an increased ‘RelB score’ compared with other clusters (Figure 1d), and increased expression of p52/RelB target genes was also identified in bulk transcriptomics, including *STAP2*, *KREMEN2*, *SOX14*, *DUSP2*, *RHOV*, *TSTD1*, *DDR1* and *LCP1* (see Table S2).

### CD45^–^ CD200^+^ CCS tumour cells express the noncanonical NF-κB signalling regulator IKKα

**Figure 2:**
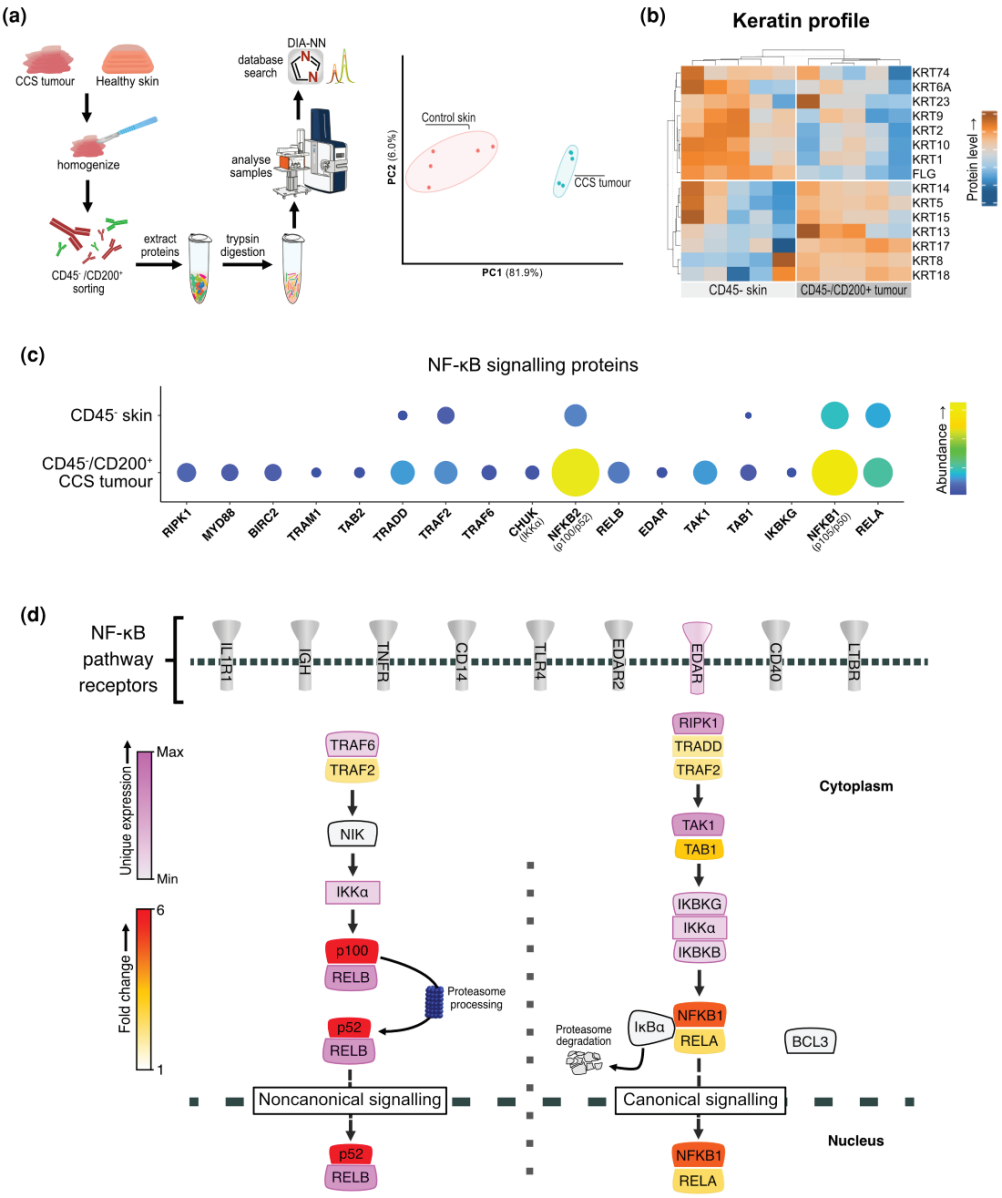
Proteomic analysis of CD45^–^ CD200^+^  *CYLD* cutaneous syndrome (CCS) tumour cells and CD45^–^ healthy skin reveals increased ectodysplasin A receptor (EDAR) and nuclear factor kappa B (NF-κB) signalling pathway members. (a) Left panel: CCS tumour and healthy skin samples were processed to enrich CD45^–^/CD200^+^ tumour cells (CCS tumour keratinocytes) and CD45^–^ skin cells (healthy keratinocytes) for proteomic analysis. Right panel: principal component (PC) analysis revealed distinct proteomes. (b) Keratin profiling of CCS tumour keratinocytes and healthy keratinocytes by hierarchical clustering of highly expressed keratin genes and filaggrin. (c) The abundance (size and colour) of NF-κB signalling proteins in CCS tumour keratinocytes compared with CD45^–^ skin cells. (d) Detected NF-κB signalling pathway proteins are projected onto a signalling diagram, showing the proteins uniquely detected in CCS tumours (pink) and the fold change increase (yellow to red). Undetected proteins in the pathway are shown in grey.

To validate our observation of deregulated noncanonical and canonical NF-κB signalling in CCS tumour keratinocytes, we characterized the CCS tumour proteome (Figure 2a). Given our patient-guided therapeutic goal of developing a treatment that targets early and preclinically apparent tumours, we sought to enrich for CCS tumour initiating cells (TICs) using CD200, a highly expressed TIC marker in CCS tumours.^4,56^ We compared CD45^–^ CD200^+^ CCS tumours (*n* = 5) and CD45^–^ enzymatically split healthy skin epidermis (*n* = 5). Protein identifications in the tumour proteome (mean = 6666) compared with healthy epidermis (mean = 1891) revealed an increased proteome complexity in CCS tumours that withstood median normalization (Figure S4; see Supporting Information) and showed differential clustering (Figure 2a). We detected 7204 proteins across all samples, with 2677 tumour-specific proteins and 3245 proteins detected in at least 3 replicates in both conditions. CD45^–^ CD200^+^ cells demonstrated increased hair follicle specific keratins (Figure 2b).

We explored the abundance of NF-κB pathway and target proteins in CD45^–^ CD200^+^ cells, detecting increased levels of TNF receptor associated factor (TRAF)2, TRAF6, CHUK/IKKα, Nuclear factor NF-kappa-B p100 subunit (NFKB2) and RelB in CCS cells compared with healthy control skin. Notably, EDAR was the only cell surface receptor known to regulate NF-κB detected, and was only detected in CCS tumours (Figure 2c, d). Canonical EDAR/NF-κB signalling was also evident, with increased expression of MAP3K7, IKBKG, IKBKB and RelA, and proteins encoded by NF-κB target genes, including BCL-2, XIAP (X-linked inhibitor of apoptosis protein) and RIPK1 (receptor-interacting serine/threonine-protein kinase 1). Additionally, in CD45^–^ CD200^+^ CCS cells, there was increased β-catenin (CTNNB1 3.14 × log2FC) (Figure S5; see Supporting Information) and reduced glycogen synthase kinase-3β (GSK3B –3.2 × log2FC), consistent with β-catenin stabilization and prior work showing nuclear β-catenin in CCS cells.^34^ EDAR overexpression in CCS alongside Wnt/β-catenin signalling is consistent with the synergy reported in luminal breast cancer.^57^

### CCS tumours and tumour spheroids demonstrate increased p100/p52 and RelB expression

**Figure 3:**
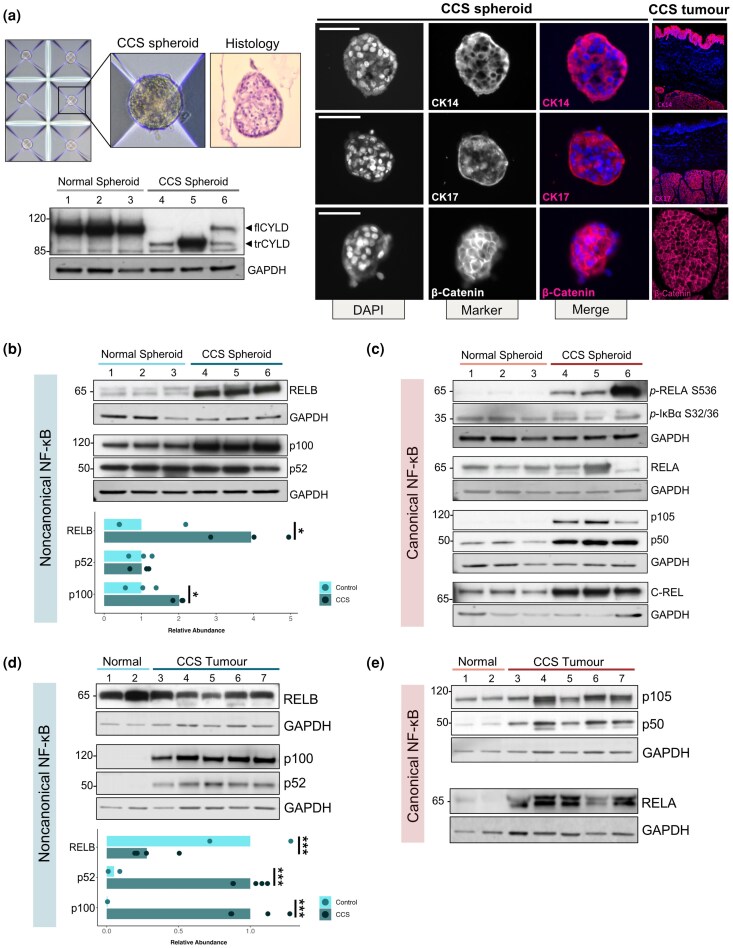
*CYLD* cutaneous syndrome (CCS) tumours and tumour spheroids show dysregulated nuclear factor kappa B (NF-κB) signalling with increased p100/p52. (a) Upper left panel: CCS tumour spheroids seen in culture and haematoxylin and eosin staining of a sectioned spheroid. Lower left panel: CCS tumour lysates show a truncated CYLD (trCYLD) protein. Right panel: CCS tumour spheroids were developed as an *in vitro* model for CCS tumours which recapitulated expression of CCS tumour markers. Immunofluorescence: images are representative of ≥ 3 spheroids, magnification × 20, scale bar = 50 μm. (b) CCS tumour spheroids represent a faithful model to study CCS tumours, with increased expression of noncanonical and (c) canonical NF-κB members. Band densitometry of replicates are plotted below the corresponding blots. (d) Expression of noncanonical and (e) canonical NF-κB members in whole tumour lysates. Band densitometry of replicates are plotted below the corresponding blot. DAPI, 4ʹ,6-diamidino-2-phenylindole; flCYLD, full-length CYLD; GAPDH, glyceraldehyde-3-phosphate dehydrogenase. *P < 0.05, *** *P* < 0.001.

To functionally investigate our findings given the lack of a mouse model, we developed a novel CCS spheroid culture model from primary tumour cells derived from surgical samples of CCS skin tumours [Figure 3a; Figure S6, Table S1 (see Supporting Information)]. CCS skin tumour spheroids were formed in Aggrewell400™ plates (STEMCELL Technologies) at calcium concentrations that facilitate keratinocyte spheroid formation without inducing differentiation.^58^ CCS keratinocyte enrichment was confirmed by truncated CYLD (trCYLD; c.2460delC) expression and absent full-length CYLD (flCYLD), and used to select samples to investigate NF-κB keratinocyte signal transduction. CCS spheroids recapitulated cytokeratin expression seen *in vivo* (Figure 3a), proliferated in culture and demonstrated evidence of reported overexpressed *in vivo* proteins, including β-catenin.^34^

The canonical and noncanonical NF-κB pathways were dysregulated in CCS spheroids (Figure 3b, c). CCS spheroids displayed increased p100 and RelB protein, and p52 levels were comparable to the control spheroids. In the canonical pathway c-Rel and p105/p50 protein were increased in CCS spheroids. Moreover, increased levels of RelA phosphorylated at S536 suggest the canonical signalling pathway is also active in tumour keratinocytes.^59^ We also investigated CCS spheroids (Figure 3b, c – lane 6) where tumour microenvironment (TME) cells persisted (evidenced by residual flCYLD; Figure 3a), finding that canonical NF-κB signalling (evidenced by increased RelA phosphorylation) was increased compared with CCS spheroids without TME cells (Figure 3b, c  **–** lanes 4, 5).

Finally, to compare the spheroids to tumours from patients with CCS, we studied the same proteins in snap-frozen CCS tumour protein lysates and healthy skin (Figure 3d, e). Here, we also found increased p100 and p52 protein and similar RelB levels in CCS tumour lysates, compared with healthy skin. Unlike healthy skin (Figure 3d, e), p100/p52 processing was already present in normal spheroids (Figure 3b, c), possibly due to the increased proliferative state in culture. Increased RelA and p50 levels were also present. These data support CCS spheroids as a faithful *in vitro* CCS model, and allowed us to study the effect of targeting noncanonical NF-κB signalling by inhibiting IKKα.

### Selective IKKα inhibition reduces CCS tumour spheroid viability and is associated with reduced truncated CYLD and p100 to p52 processing

**Figure 4:**
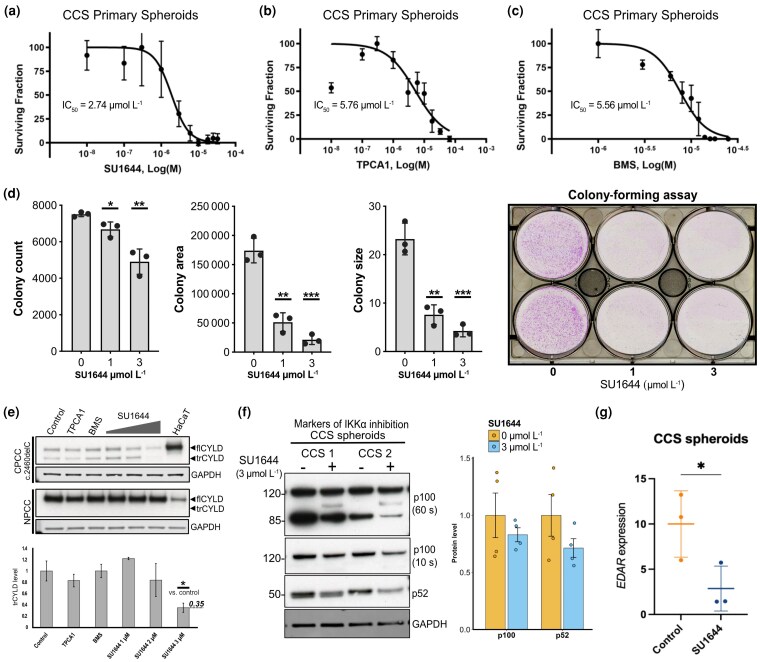
Targeting IκB kinase alpha (IKKα) with the highly selective IKKα inhibitor SU1644 results in reduced *CYLD* cutaneous syndrome (CCS) spheroid viability, with reduced truncated CYLD (trCYLD) and EDAR. CCS spheroid dose–response to small-molecule IKK inhibitors assessed by a CellTiter-Glo^®^ Luminescent Cell Viability Assay (Promega, Madison, WI, USA) after treatment with (a) SU1644 targeting IKKα, (b) TPCA-1 targeting IκB kinase beta (IKKβ) or (c) BMS-345541 (BMS) targeting IKKα and IKKβ. All graphs show the mean (SEM). (d) Left panel: CCS primary cells (CPCC) were seeded and cultured in the presence of 0, 1 or 3 μmol L^–1^ SU1644 and the count, area and size of colonies were quantified after 10 days in culture. Right panel: colony-forming assay plate at day 10. (e) Levels of trCYLD and full-length CYLD (flCYLD) in CPCC and normal primary cell culture (NPCC) detected by immunoblotting after 24 h of treatment with IKK inhibitors. HaCaT lysate is included as a positive control for flCYLD. TPCA-1 and BMS were used at a concentration of 6 μmol L^–1^. (f) Left panel: p100 to p52 is reduced in CCS tumour spheroids after 24 h of SU1644 treatment. Right panel: band densitometry of four biological replicates. (g) *EDAR* mRNA expression measured by real-time quantitative polymerase chain reaction is reduced in CCS spheroids following 24 h of treatment with 3 μmol L^–1^ SU1644. *P < 0.05, **P < 0.01, ***P < 0.001 (Student’s *t*-test). GAPDH, glyceraldehyde-3-phosphate dehydrogenase.

IKKα is upregulated in CD45^–^ CD200^+^ CCS tumour cells (Figure 2c), and is not itself shown to be mutated in CCS.^3^ Therefore, selectively targeting IKKα could reduce noncanonical NF-κB signalling as a novel therapeutic strategy for CCS. As part of an ongoing drug discovery programme to develop selective IKKα inhibitors that demonstrate low affinity for IKKβ, we have developed SU1644 (patent: PCT/GB2023/051242). SU1644 demonstrated selective IKKα target engagement in a metastatic prostate cancer cell line (PC3m) using pharmacodynamic markers [p100 phosphorylation; RelB nuclear translocation (IC_50_ = 0.05 µmol L^–1^)], with no activity against IKKβ markers evident at 10 µmol L^–1^ (data not shown). SU1644 also had a highly selective kinase profile when assessed against a representative kinome sample (Figure S7; see Supporting Information). Topical delivery of SU1644 itself is attractive because it is a relatively lipophilic compound having a high log P (3.6) with high hepatocyte clearance, (220 μL min^–1^ 10^6^ cells; t_1/2_= 6 min), which would lead to low systemic exposure and thus avoid perturbation of noncanonical NF-κB signalling in other tissues (data not shown). We used SU1644 treatment in our CCS spheroid model and found an IC_50_ of 2.74 μmol L^–1^ (Figure 4a), without the induction of apoptosis at these concentrations, as measured by cleaved caspase 3 levels (Figure S8; see Supporting Information). We targeted IKKβ specifically with TPCA-1 (Figure 4b), and both IKKα and IKKβ with BMS-345541 (IC_50_ values are 0.3 and 4.0 μmol L^–1^ for IKKβ and IKKα, respectively) (Figure 4c), which both showed higher IC_50_ values (5.76 μmol L^–1^ and 5.56 μmol L^–1^, respectively) in our CCS spheroid model. Additionally, we cultured CCS tumour primary cells in the presence of 0, 1 or 3 μmol L^–1^ SU1644 in a colony-forming assay (Figure 4d). We counted statistically significantly fewer colonies, with a smaller area and size with increasing concentrations of SU1644, indicating that SU1644 reduces the proliferation of CCS cells.

We studied the effect of selective IKKα inhibition with SU1644 on trCYLD in CCS. SU1644 (3 μmol L^–1^) reduced trCYLD levels in CPCC cultures from patients with a truncating PV and a splice site PV [Figure 4e; Figure S9 (see Supporting Information)]. SU1644 also reduced p100 processing and p52 levels, consistent with a decrease in noncanonical NF-κB signalling (Figure 4f). CCS spheroid *EDAR* expression was also significantly reduced after SU1644 treatment, confirming its regulation by the noncanonical NF-κB pathway indicated above (Figure 4g). Together, these findings identify IKKα inhibition as a novel candidate for CCS tumour prevention.

## Discussion

CCS is a progressive condition that lacks medical therapies. Mechanistic understanding of oncogenic drivers in tumour keratinocytes is a prerequisite for the development of novel treatments. By studying samples from patients with CCS using complementary transcriptomic and proteomic methods, we have demonstrated deregulated noncanonical and canonical NF-κB signalling in CCS tumour keratinocytes. We demonstrated increased p100 and p52 levels *in vivo* – a hallmark of noncanonical signalling – highlighting NIK and IKKα, which mediate p100 processing, as attractive druggable targets.^60^ Here, we demonstrate that the selective targeting of IKKα with SU1644 in the noncanonical NF-κB pathway reduces *EDAR* expression and cell viability in patient-derived CCS spheroids.

EDAR overexpression is shown to synergize with increased β-­catenin signalling in mouse mammary cancer models towards driving tumorigenesis.^57^ Precise spatiotemporal expression of EDAR/NF-κB signalling in human development is crucial for hair follicle, skin appendage and tooth development.^61^ Loss-of-function mutations in *EDA* or *EDAR* lead to a reduction in EDAR/NF-κB signalling and ectodermal dysplasia phenotypes with absent hair, sweat glands and teeth.^62,63^ We propose that together with activated Wnt/β-catenin signalling,^33,34^ EDAR signalling in CCS promotes CCS tumour keratinocyte viability. Expression of antiapoptotic targets of canonical NF-κB signalling such as BCL-2, previously described in these tumours,^2^ suggests that the progressive growth that results in the exceptionally large CCS tumours may be mediated by apoptosis resistance.^64^

Delineation of the events that lead to increased p100/p52 processing remains to be determined, but we provide data that implicate a hypomorphic trCYLD protein. Murine studies have established that the phenotype associated with CYLD truncation or expression of alternate short isoforms is distinct from that seen in CYLD ablation.^22^ In humans, CCS tumours are predominantly driven by truncating mutations, suggesting the hypomorphic protein in the absence of flCYLD is a key driver. trCYLD expression was reduced following IKKα inhibition, which may be related to the role of IKK in regulating mRNA stability.^65,66^ trCYLD reduction may also reflect selective killing of CCS tumour keratinocytes by SU1644, and the IC_50_ for tumour cells may be lower than reported (Figure 4a). Taken together, we suggest that trCYLD interferes with regulation of noncanonical NF-κB signalling. Given the multiple targets of CYLD, this is likely mediated by more than one substrate. It remains to be understood how the expressed CYLD protein in infrequent missense *CYLD* PV carriers abrogates signalling.^1^ Recently, work to understand the crystal structure of predicted truncated variants suggests that analysis of tertiary protein structures may reveal how conformational changes from missense PVs may also impact on NF-κB signalling.^67^

Increased noncanonical and canonical NF-κB signalling in putative tumour initiating CD200^+^ CD45^–^ CCS cells is especially relevant in the development of ‘pre-emptive’ topical treatments applied to at-risk skin for patients with CCS, as IKKα inhibition may potentially eliminate preclinically apparent CCS tumour cells. CCS is suitable for topical delivery of IKKα inhibitors, circumventing the potential side effects seen with systemic delivery of IKKβ inhibitors.^68^ Further work is necessary in preclinical models to study the *in vivo* effects of IKKα inhibition on the tumour immune response (Figure S2b). Together, these data support the investigation of further preclinical studies to determine the efficacy and safety of selective IKKα inhibition as a strategy to reduce the tumour burden and need for surgical intervention for patients living with CCS.

## Supplementary Material

ljag044_Supplementary_Data

## Data Availability

The data underlying this article will be shared on reasonable request to the corresponding author.
