# Supplementary material for: Targeting noncanonical nuclear factor kappa B signalling in CYLD cutaneous syndrome by selective inhibition of IκB kinase alpha
Source: Br J Dermatol. 2026 Mar 4;194(6):1130–41. doi: 10.1093/bjd/ljag044 (PMC13185748; doi:10.1093/bjd/ljag044)
Supplement: ljag044_Supplementary_Data [file ljag044_supplementary_data.pdf]

Supplementary Materials and Methods

Cell culture

CCS tumours were collected in Dulbecco's modified eagle medium (DMEM) with 10% foetal bovine serum (FBS) immediately after surgery, transported to a class II cell culture hood, cut into ~1mm^3 pieces and transferred into 15 ml centrifuge tubes. One 75 cm^2 flask per tumour was coated with collagen (R011K Gibco) and incubated at room temperature during sample preparation. After centrifugation at 900 x g and decanting the supernatant, tumours were digested in 5 ml 2.5% trypsin for 40 min at 37°C with gentle agitation every 20 min. Tumours were centrifuged at 900 x g to allow aspiration of 3 ml trypsin. The pellets were incubated in 5 ml collagenase (1 mg/ml) for 70 min at 37°C with gentle agitation every 20 min. The dissociated tumour suspensions were transferred to 50 ml centrifuge tubes and 3 volumes of DMEM added before passing through a 40 um filter into a new 50 ml tube. Each quarter of the cell suspension was transferred to a 15 ml centrifuge tube and centrifuged at 900 x g. The resulting pellet was resuspended in complete defined keratinocyte serum free medium (DKSFM Gibco 10744019)/0.2% penicillin-streptomycin (5000 units/ml)/1% L-glutamine (200 mM)/0.04% Amphotericin B (250 µg/ml) and seeded in a collagen coated 75 cm^2 flask for continued culturing. Alternatively, the pellet was resuspended 10% dimethylsulfoxide (DMSO)/FBS and cryopreserved at -80°C. Cell cultures were maintained in complete DKSFM supplemented with 10 µM rho kinase inhibitor (Y-27632 Tocris) and passaged when ~80% confluent.

Normal skin primary cell culture

Immediately after surgery for non-CCS related conditions, donated skin was collected in complete DMEM with Dispase II (1 unit/ml) and incubated on a roller at 4°C overnight. One 25 cm^2 flask per sample was coated with collagen (R011K Gibco) and incubated at room temperature during sample preparation. The epidermis was peeled from the dermis with fine forceps, transferred to a 15 ml centrifuge tube containing 5 ml 0.05% Trypsin-EDTA and incubated for 30 min at 37°C with gentle agitation by pipetting every 15 minutes. After adding 5 ml complete DMEM and centrifugation at 300 x g for 5 min, the supernatant was aspirated and the pellet resuspended in complete Epilife medium (Gibco MEPI500CA)/0.2% penicillin-streptomycin (5000 U/ml)/1% L-glutamine (200 mM)/0.04% Amphotericin B (250 µg/ml). Cells were seeded onto collagen coated flasks and passaged when 80% confluent. Cells were maintained in complete Epilife medium supplemented with 10 µM Rho kinase inhibitor (Y-27632 Tocris).

2D Cell passage and harvesting for protein

Cells were passaged by washing with phosphate-buffered saline (PBS), incubating in versene (Gibco 15040-066) for 5 min at 37°C followed by 0.05% Trypsin-EDTA at 37°C until dissociated. After quenching with complete DMEM, cells were centrifuged at 900 x g and resuspended in 1 ml PBS. Flasks were reseeded with 20% of the cell suspension and the remaining cells were transferred to a microcentrifuge and centrifuged at 17,000 x g for protein extraction. The supernatant was completely aspirated and pellets were frozen on dry ice immediately and stored at -80°C.

Harvesting spheroids for protein

On day 4 of spheroid culture, ¾ media was removed from each well and the remaining media pipetted firmly to dislodge the spheroids from the microwells. The spheroid containing media was passed through a 40 um filter into a 50 ml tube to remove single cells. Cell culture medium (1 ml) supplemented with 1.5 mM $CaCl_2$ and 5 mM EDTA was used to wash each well twice and each wash was passed through the filter. The filter was then inverted and spheroids were recovered by rinsing with 5 ml media/1.5 mM $CaCl_2$/5 mM EDTA into a new tube. The spheroid suspension was centrifuged at 2000 x g for 5 min and the media aspirated. The resulting pellet was frozen immediately on dry ice and stored at -80°C for downstream protein extraction.

Cell viability assay

Primary cells were seeded in 96 well spheroid microplates (Corning 4520) at 30,000 per well in 280 µl complete media supplemented with 1.5 mM CaCl2. After 3-7 days, spheroids were drugged at the range of concentrations indicated. After 72 hours incubation with TPCA1/BMS345541 or 48 hours incubation with SU1644, cell viability was assessed with the CellTiter-Glo® Luminescent Cell Viability Assay (Promega). Luminescence was recorded with the Varioskan™ LUX microplate reader. Dose response curves were generated using GraphPad PRISM version 9. Error bars are standard deviation of the mean of three replicates wells for each concentration. Three biological repeats were performed for each experiment, with representative data and mean IC_{50} values shown in the figures.

Protein extraction

Tissue curls were cut at 30 µm on a cryostat from snap frozen CCS tumour or normal skin. Tissue was lysed in 150 µl Novagen's PhosphoSafe™ Extraction Reagent (EMD Millipore) or 8M Urea lysis buffer (8M Urea, 50 mM Tris pH 8, 300 mM NaCl, 50 mM Na2HPO4, 0.5% NP-40) and homogenised in Precellys® CK Mix 2 ml bead tubes. Samples were centrifuged in the bead tubes at 4°C, 15,000 x g for 5 min to reduce bubbles then transferred to microcentrifuge tubes. After centrifuging again at 4°C for 15 min at 15,000 x g, samples were sonicated 3 times for 15 s at 30% amplitude. Samples were centrifuged at 4°C for 20 min at 15,000 x g and the supernatant was transferred to a new microcentrifuge tube. Protein was extracted from spheroid and 2D cell pellets by sonication for 3 x 15 s at 30% amplitude in 40–100 µl 8M Urea lysis buffer. Protein concentration was measured with the Pierce™ BCA protein assay kit.

Proteomics analysis

FACS sorted samples were lysed in 5% SDS, 8 M urea, 100 mM glycine pH 7.55 followed by concurrent reduction with 20 mM tris (2-carboxyethyl) phosphine hydrochloride (TCEP) and alkylation with 20 mM Iodoacetamide for 30 min at 37 °C in the dark. Samples were acidified with phosphoric acid to 1% v/v. Proteins were precipitated with 90% Methanol, 10% TEAB v/v, and the resulting mixture added to ProtiFi micro S-Trap column. Samples were passed through the column by centrifugation at 4,000 x g for 1 min and washed with 90% Methanol, 10% TEAB v/v 5 times by centrifugation. Column bound proteins

were digested with 1:20 trypsin at 47 °C for 2 h. Resulting peptides were serially eluted with 40 µL of 50 mM TEAB pH 8.0, 0.2% formic acid (FA) in water and 0.2% FA in 50% acetonitrile, 50% water. Eluates were dried by vacuum concentration and resuspended in 0.1% FA in water.

500 ng of digest was analysed on a Bruker TIMS-TOF HT mass spectrometer in line with the EvoSep One LC system, as previously reported.[69] Samples were separated by elution in a gradient of solvent A (0.1 % FA in HPLC water) and solvent B (0.1 % FA in HPLC acetonitrile), by the Evosep One Whisper 20 samples per day method. The mass spectrometer was operated in positive-ion, data independent acquisition parallel accumulation serial fragmentation (DIA-PASEF) mode. Precursor ion scans (full scan, MS1) were in the range of 100-1,700 *m/z*, while DIA-PASEF (MS2 scans), mass and IM ranges were 300-1200 *m/z* and 0.6-1.4 $1/K_0$, with 16 variable width IM-*m/z* windows, designed using py_diAID.[70] Collision energy was applied as a linear gradient from 20-59 eV to precursors with mobilities from 0.60-1.60 V.s/cm2. Ramp and accumulation time were 100 ms, with a ramp rate of 9.42 Hz. Total cycle time was ~1.8 sec. Data files were searched against an in-silico digest of SwissProt *Homo sapiens* database using DIA-NN 1.8.1.[71]

Immunoblotting

Samples were prepared with NuPAGE™ lithium dodecyl sulphate (LDS) loading buffer and dithioreitol (DTT) sample reducing agent (Invitrogen) to 0.5 µg/µl protein. Samples were heated at 70°C for 10 min, loaded on NuPAGE Tris-Acetate or Bis-Tris precast gels in the appropriate running buffer (NuPAGE Tris-Acetate, MES or MOPS sodium dodecyl sulphate (SDS) running buffer, Invitrogen) and separated with the NuPAGE gel electrophoresis system before transfer onto PVDF membranes with the Bio-Rad Transblot Turbo semi-dry transfer system. PVDF membranes were blocked in Tris buffered saline with Tween-20 detergent (TBST) (20 mM Tris pH 7.6, 120 mM NaCl, 0.1% Tween 20) and 5% w/v fat-free milk or 3% w/v BSA for 1 h. Membranes were subsequently incubated in primary antibody solution overnight at 4°C and secondary antibody conjugated to horseradish peroxidase (HRP) (Cell signalling) or a fluorescent dye for 1 h at room temperature (see Table 2-8 for antibodies). Washes between incubations were performed in TBST for 3 x 10 min. HRP enzyme activity was detected with enhanced chemiluminescent (ECL) substrate. Fluorescence was detected with the LICOR Odyssey® CLx imaging system.

Antibodies used for immunoblotting. CST, Cell Signaling Technology; Thermo, ThermoFisher Scientific; Phospho, phosphorylated.

Antibody	Supplier	Cat. No.	Dilution
RelA	CST	4764	1:1000
Phospho RelA serine 536	CST	3031S	1:1000
RelB	CST	4954	1:1000
c-Rel	CST	67489	1:1000
p100/p52	CST	3017	1:1000
p105/p50	05-361	Millipore	1:1000
CYLD	CST	12797	1:1000

Phospho IκBα serines 32/36	CST	9246	1:1000
EDAR	Protein Tech	18032-1-AP	1:1000
GAPDH (mouse)	CST	97166	1:1000
GAPDH (rabbit)	CST	2118	1:1000
**Secondary antibodies**			
Goat anti-rabbit HRP linked	CST	7074	1:5000
Horse anti-mouse HRP linked	CST	7076	1:5000
IRDye® 680RD donkey anti-mouse	LI-COR	925-68072	1:5000
Alexa Fluor® 790 donkey anti-rabbit	Thermo	A11374	1:5000

Immunofluorescence

Tissue sections from snap frozen skin tumours were fixed in ice cold methanol for 10 min, washed in PBS for 3 x 10 min, 30% sucrose in PBS w/v for 30 min and then blocked in 0.5% BSA, 0.3% Triton X-100 w/v in PBS for 1 h at room temperature. Immunofluorescent labelling with primary antibodies against CYLD, CK14, CK17, CK18, β-catenin and Ki67 was performed overnight at 4°C. The following day, sections were washed with 0.2% BSA, 0.1% Triton X-100 w/v in PBS for 3 x 10 min. DAPI (4′,6-diamidino-2-phenylindole) nuclear stain (0.1 µg/ml) and secondary fluorophore conjugated antibodies were applied for 1 h at room temperature in the dark. After washing 3 x 10 minutes with 0.2% BSA, 0.1% Triton X-100 w/v in PBS, coverslips were mounted with ProLong Gold Antifade (Thermofisher Scientific) and fluorescence images captured by structured illumination microscopy using the Zeiss Axioimager Z2, with Apotome 2.

Antibodies used for immunofluorescence. CST, Cell Signaling Technology; BDT, BD Transduction; Jackson, Jackson ImmunoResearch.

Antibody	Supplier	Cat. No.	Dilution
**Primary antibodies**			
CK14	Abcam	Ab7800	1:800
CK17	Abcam	Ab19067	1:100
CK18	Abcam	Ab668	1:200
β-catenin	BDT	610153	1:50
Ki67	CST	9449	1:400
**Secondary antibodies**			
Alexa Fluor® 488 goat anti-rabbit	Jackson	111-545-144	1:200
Alexa Fluor® 594 goat anti-mouse	Jackson	115-585-146	1:400
Alexa Fluor® 594 donkey anti-guinea pig	Jackson	706-585-148	1:400

Fluorescence activated cell sorting

Tissue was cut into 1 mm^3 pieces and digested by incubation in 1.5 mg/ml collagenase (Sigma C1889) for 2 h at 37°C with gentle agitation by pipetting every 20 min. DNase I (Sigma 10104159001) was

added after one hour at a final concentration of 1 mg/ml. After resuspension in flow buffer (5mM EDTA, 2% FBS w/v in PBS) and passing through a 40µm filter, cells were centrifuged at 900 x g for 5 min and the supernatant aspirated. Red blood cells were removed by resuspending the pellet in red blood cell lysis buffer (Abcam ab204733) and incubating at room temperature for 10 min. Remaining cells were centrifuged at 900 x g for 5 min, resuspended in 2 ml flow buffer and counted. 500 µl was taken from each sample for RNAseq and kept on ice. The remaining cell suspension was centrifuged at 900 x g for 5 min and the flow buffer aspirated leaving 50 – 100 µl covering the pellet. Cells were resuspended and stained with CD45 antibody conjugated to fluorescein isothiocyanate (FITC) (Thermo Fisher 11-9459-42) at 5µl/10^5 - 10^8 cells in the dark at 4°C for 30 min. During this incubation, the input fractions were centrifuged at 20,000 x g for 3 min, resuspended in 350 µl ice cold RLT buffer from Qiagen's RNeasy Micro Kit (74004) with added 1% 2-Mercaptoethanol and frozen on dry ice. CD45 stained cells were topped up with cold flow buffer to 5 ml and centrifuged at 900 x g for 5 min, resuspended in flow buffer at a maximum of 1 x 10^7/ml and transferred to a FACS tube. DAPI was added at a final concentration of 3 µM immediately prior to analysis by flow cytometer. Cells were sorted with the BD FACSAria™ II flow cytometer into 200µl FBS. Sorted cells were centrifuged at 4°C for 3 min at 20,000 x g, resuspended in 350µl RLT buffer with 1% 2-Mercaptoethanol and frozen on dry ice. Samples were stored at -80°C in RLT buffer for downstream RNA extraction with the RNeasy Micro Kit (Qiagen 74004) following the manufacturer's protocol.

Statement regarding sex as a biological variable

Our study examined male and female samples. Similar findings are reported for both sexes.

References

69 Hermosilla-Trespaderne M, Hu-Yang MX, Dannoura A, *et al.* Proteomic Analysis Reveals Trilaciclib-Induced Senescence. *Mol Cell Proteomics* 2024; :100778.

70 Skowronek P, Thielert M, Voytik E, *et al.* Rapid and In-Depth Coverage of the (Phospho-)Proteome With Deep Libraries and Optimal Window Design for dia-PASEF. *Mol Cell Proteomics* 2022; **21**:100279.

71 Demichev V, Messner CB, Vernardis SI, *et al.* DIA-NN: neural networks and interference correction enable deep proteome coverage in high throughput. *Nat Methods* 2020; **17**:41–4.

Supplementary Table Legends

Supplementary Table 1. Patient samples used in this study.

Supplementary Table 2. Candidate non-canonical NF-κB target genes differentially expressed between CD45- CCS tumour and normal cells.

Supplementary Table 1 - Samples used in this study.

Patient No	CYLD Genotype	Sex	Age at biopsy	Tissue source	Processing	Assay	Assay sample no
1	WT	M	79	Normal skin	Input	RNAseq	1
1	WT	M	79	Normal skin	CD45 positive	RNAseq	2
1	WT	M	79	Normal skin	CD45 negative	RNAseq	3
2	WT	M	77	Normal skin	Input	RNAseq	4
2	WT	M	77	Normal skin	CD45 positive	RNAseq	5
2	WT	M	77	Normal skin	CD45 negative	RNAseq	6
3	c.2460delC	F	77	Cylindroma	Input	RNAseq	7
3	c.2460delC	F	77	Cylindroma	CD45 positive	RNAseq	8
3	c.2460delC	F	77	Cylindroma	CD45 negative	RNAseq	9
4	c.2460delC	F	64	Cylindroma	Input	RNAseq	10
4	c.2460delC	F	64	Cylindroma	CD45 positive	RNAseq	11
4	c.2460delC	F	64	Cylindroma	CD45 negative	RNAseq	12
5	c.2460delC	F	73	Spiradeno ma	Bulk tissue	RNAseq	13
5	c.2460delC	F	78	Cylindrospir adenoma	Bulk tissue	RNAseq	14
5	c.2460delC	F	78	Cylindroma	Bulk tissue	RNAseq	15
6	c.2460delC	F	55	Cylindroma	Input	RNAseq	16
6	c.2460delC	F	55	Cylindroma	CD45 positive	RNAseq	17
6	c.2460delC	F	55	Cylindroma	CD45 negative	RNAseq	18
7	WT	F	67	Normal skin	Input	RNAseq	19
7	WT	F	67	Normal skin	CD45 positive	RNAseq	20
7	WT	F	67	Normal skin	CD45 negative	RNAseq	21
7	WT	F	67	Normal skin	Input	RNAseq	22
7	WT	F	67	Normal skin	CD45 positive	RNAseq	23
7	WT	F	67	Normal skin	CD45 negative	RNAseq	24
8	c.2460delC	F	50	Cylindroma	Input	RNAseq	25
8	c.2460delC	F	50	Cylindroma	CD45 positive	RNAseq	26
8	c.2460delC	F	50	Cylindroma	CD45 negative	RNAseq	27
8	c.2460delC	F	50	Cylindroma	Input	RNAseq	28
8	c.2460delC	F	50	Cylindroma	CD45 positive	RNAseq	29
8	c.2460delC	F	50	Cylindroma	CD45 negative	RNAseq	30
9	WT	F	83	Normal skin	Bulk tissue	RNAseq	31
10	WT	M	29	Normal skin	Bulk tissue	RNAseq	32
11	WT	M	78	Normal skin	Bulk tissue	RNAseq	33
8	c.2460delC	F	50	Cylindroma	Bulk tissue	RNAseq	34
8	c.2460delC	F	50	Cylindroma	Bulk tissue	RNAseq	35
12	c.2469+1A>G	F	82	Cylindroma	Bulk tissue	RNAseq	36
12	c.2469+1A>G	F	82	Cylindrospir adenoma	Bulk tissue	RNAseq	37
3	c.2460delC	F	77	Cylindroma	Bulk tissue	RNAseq	38
5	c.2460delC	F	73	Spiradeno ma	Bulk tissue	RNAseq	39

Supplementary Table 1 - Samples used in this study.

Patient No	CYLD Genotype	Sex	Age at biopsy	Tissue source	Processing	Assay	Assay sample no
5	c.2460delC	F	73	Cylindroma	Bulk tissue	RNAseq	40
11	WT	M	78	Normal skin	Tissue	Immunoblot (RelA, p100/p52)	1
11	WT	M	78	Normal skin	Tissue	Immunoblot (RelA, p100/p52)	2
13	c.2460delC	F	63	Cylindroma	Tissue	Immunoblot (RelA, p100/p52)	3
8	c.2460delC	F	47	Cylindroma	Tissue	Immunoblot (RelA, p100/p52)	4
3	c.2460delC	F	71	Cylindroma	Tissue	Immunoblot (RelA, p100/p52)	5
13	c.2460delC	F	63	Cylindroma	Tissue	Immunoblot (RelA, p100/p52)	6
8	c.2460delC	F	47	Cylindroma	Tissue	Immunoblot (RelA, p100/p52)	7
11	WT	M	78	Normal skin	Tissue	Immunoblot (RelB, p105/p50)	1
11	WT	M	78	Normal skin	Tissue	Immunoblot (RelB, p105/p50)	2
8	c.2460delC	F	39	Cylindroma	Tissue	Immunoblot (RelB, p105/p50)	3
8	c.2460delC	F	47	Cylindroma	Tissue	Immunoblot (RelB, p105/p50)	4
3	c.2460delC	F	71	Cylindroma	Tissue	Immunoblot (RelB, p105/p50)	5
13	c.2460delC	F	63	Cylindroma	Tissue	Immunoblot (RelB, p105/p50)	6
8	c.2460delC	F	47	Cylindroma	Tissue	Immunoblot (RelB, p105/p50)	7
13	c.2460delC	F	61	Cylindroma	2D CPCC4	Immunoblot (CYLD shRNA)	1
14	c.2460delC	F	44	Cylindroma	2D CPCC2	Immunoblot (CYLD shRNA)	2
15	WT	M	94	Normal skin	Tissue	CD45- FACS and proteomics	*C1*
16	WT	M	83	Normal skin	Tissue	CD45- FACS and proteomics	*C2*
17	WT	M	69	Normal skin	Tissue	CD45- FACS and proteomics	*C3*
18	WT	F	68	Normal skin	Tissue	CD45- FACS and proteomics	*C4*
19	WT	F	55	Normal skin	Tissue	CD45- FACS and proteomics	*C5*
20	WT	F	67	Cylindroma	Tissue	CD45-CD200+ enrichment and proteomics	*T1*
21	c.2460delC	F	54	Cylindroma	Tissue	CD45-CD200+ enrichment and proteomics	*T2*
8	c.2460delC	F	54	Cylindroma	Tissue	CD45-CD200+ enrichment and proteomics	*T3*
22	Sporadic	F	73	Cylindroma	Tissue	CD45-CD200+ enrichment and proteomics	*T4*
8	c.2460delC	F	54	Cylindroma	Tissue	CD45-CD200+ enrichment and proteomics	*T5*
23	WT	M	56	Normal skin	Tissue	Immunoblot (CYLD)	1
11	WT	M	78	Normal skin	Tissue	Immunoblot (CYLD)	2
24	c.1112C>A	M	39	Cylindroma	Tissue	Immunoblot (CYLD)	3
25	5.5MB deletion	F	54	Cylindroma	Tissue	Immunoblot (CYLD)	4
25	5.5MB deletion	F	54	Cylindroma	Tissue	Immunoblot (CYLD)	5
8	c.2460delC	F	49	Cylindroma	Tissue	Immunoblot (CYLD)	6
26	c.2469+1G>A	M	75	Cylindroma	Tissue	Immunoblot (CYLD)	7
27	WT	M	71	Normal skin	Spheroids	Immunoblot (NF-kB)	1
28	WT	M	83	Normal skin	Spheroids	Immunoblot (NF-kB)	2
29	WT	M	68	Normal skin	Spheroids	Immunoblot (NF-kB)	3
8	c.2460delC	F	46	Cylindroma	Spheroids	Immunoblot (NF-kB)	4
30	c.2469+1G>A	M	51	Cylindroma	Spheroids	Immunoblot (NF-kB)	5
14	c.2460delC	F	44	Cylindroma	Spheroids	Immunoblot (NF-kB)	6
29	WT	M	68	Normal skin	2D NPCC	Immunoblot (CYLD, RelB, Edar)	1
31	WT	M	71	Normal skin	2D NPCC	Immunoblot (CYLD, RelB, Edar)	2
3	c.2460delC	F	76	Cylindroma	2D CPCC	Immunoblot (CYLD, RelB, Edar)	3
14	c.2460delC	F	44	Cylindroma	2D CPCC	Immunoblot (CYLD, RelB, Edar)	4
32	WT	M	71	Normal skin	Spheroids	Immunoblot (CYLD, RelB, Edar)	5
33	WT	M	83	Normal skin	Spheroids	Immunoblot (CYLD, RelB, Edar)	6
8	c.2460delC	F	46	Cylindroma	Spheroids	Immunoblot (CYLD, RelB, Edar)	7
30	c.2469+1G>A	M	51	Cylindroma	Spheroids	Immunoblot (CYLD, RelB, Edar)	8

Supplementary Table 1 - Samples used in this study.

Patient No	CYLD Genotype	Sex	Age at biopsy	Tissue source	Processing	Assay	Assay sample no
3	c.2460delC	F	76	Cylindroma	Spheroids	Viability (TPCA1)	1
5	c.2460delC	F	81	Cylindroma	Spheroids	Viability (TPCA1)	2
30	c.2469+1G>A	M	51	Cylindroma	Spheroids	Viability (TPCA1)	3
5	c.2460delC	F	81	Cylindroma	Spheroids	Viability (BMS)	1
30	c.2469+1G>A	M	51	Cylindroma	Spheroids	Viability (BMS)	2
30	c.2469+1G>A	M	51	Cylindroma	Spheroids	Viability (BMS)	3
14	c.2460delC	F	44	Cylindroma	Spheroids	Viability (SU1644)	1
3	c.2460delC	F	76	Cylindroma	Spheroids	Viability (SU1644)	2
30	c.2469+1G>A	M	51	Cylindroma	Spheroids	Viability (SU1644)	3
30	c.2469+1G>A	M	51	Cylindroma	2D CPCC2	Immunoblot (CYLD +inhibitors)	1
3	c.2460delC	F	76	Cylindroma	2D CPCC3	Immunoblot (CYLD +inhibitors)	2
14	c.2460delC	F	44	Cylindroma	2D CPCC4	Immunoblot (CYLD +inhibitors)	3
29	WT	M	68	Normal skin	2D NPCC1	Immunoblot (CYLD +inhibitors)	4
29	WT	M	68	Normal skin	2D NPCC2	Immunoblot (CYLD +inhibitors)	5
31	WT	M	71	Normal skin	2D NPCC3	Immunoblot (CYLD +inhibitors)	6
34	WT	F	61	Normal skin	Spheroids	Immunoblot (p100/p52 +SU1644)	1
35	WT	M	83	Normal skin	Spheroids	Immunoblot (p100/p52 +SU1644)	2
30	c.2469+1G>A	M	51	Cylindroma	Spheroids	Immunoblot (p100/p52 +SU1644)	3
13	c.2460delC	F	61	Cylindroma	Spheroids	Immunoblot (p100/p52 +SU1644)	4
8	c.2460delC	F	53	Cylindroma	CD45 negative	scRNAseq	1
8	c.2460delC	F	53	Cylindroma	CD45 negative	scRNAseq	2
8	c.2460delC	F	53	Cylindroma	CD45 negative	scRNAseq	3

Supplementary Table 2. Candidate non-canonical NF-κB target genes differentially expressed between CD45$^-$ normal skin and CCS tumour cells.

No.	log_2FC	Gene	Description
1	8.2	*EDAR*	Ectodysplasin A receptor
2	8.0	*SOX14*	SRY-box 14
3	5.4	*KREMEN2*	Kringle containing transmembrane protein 2
4	5.2	*CD164L2*	CD164 molecule like 2
5	5.2	*PLA2G4D*	Phospholipase A2 group IVD
6	5.0	*LCP1*	Lymphocyte cytosolic protein 1
7	5.0	*SOX21*	SRY-box 21
8	4.1	*COL22A1*	Collagen type XXII alpha 1 chain
9	4.0	*FAM71E1*	Family with sequence similarity 71 member E1
10	3.9	*RHOV*	Ras homolog family member V
11	3.8	*FAM83F*	Family with sequence similarity 83 member F
12	3.5	*KY*	Kyphoscoliosis peptidase
13	3.5	*FAAH2*	Fatty acid amide hydrolase 2
14	3.4	*FFAR2*	Free fatty acid receptor 2
15	3.2	*TSTD1*	Thiosulfate sulfurtransferase like domain containing 1
16	3.2	*DDR1*	Discoidin domain receptor tyrosine kinase 1
17	3.2	*DUSP2*	Dual specificity phosphatase 2
18	3.1	*ANO9*	Anoctamin 9
19	3.1	*ANK3*	Ankyrin 3
20	2.9	*KCNB2*	Potassium voltage-gated channel subfamily B member 2
21	2.9	*VAX2*	Ventral anterior homeobox 2
22	2.9	*ELF3*	E74 like ETS transcription factor 3
23	2.6	*LHFPL4*	LHFPL tetraspan subfamily member 4
24	2.5	*THSD7B*	Thrombospondin type 1 domain containing 7B
25	2.5	*NMBR*	Neuromedin B receptor
26	2.5	*STAP2*	Signal transducing adaptor family member 2
27	2.5	*HEPACAM*	Hepatic and glial cell adhesion molecule
28	2.4	*ZNF385C*	Zinc finger protein 385C
29	2.4	*DAPP1*	Dual adaptor of phosphotyrosine and 3-phosphoinositides 1
30	2.4	*TRAF1*	TNF receptor associated factor 1
31	2.4	*SAMD5*	Sterile alpha motif domain containing 5
32	2.4	*ARHGAP4*	Rho GTPase activating protein 4
33	2.3	*BSN*	Bassoon presynaptic cytomatrix protein
34	2.3	*SGPP2*	Sphingosine-1-phosphate phosphatase 2
35	2.2	*GOLT1A*	Golgi transport 1A
36	2.2	*AGBL2*	ATP/GTP binding protein like 2
37	2.1	*GNA15*	G protein subunit alpha 15
38	2.1	*HPCA*	Hippocalcin
39	2.0	*RNF227*	Ring finger protein 227
40	-2.0	*CLGN*	Calmegin
41	-2.1	*HTRA1*	HtrA serine peptidase 1

No.	log_2FC	Gene	Description
42	-2.2	*CLDN5*	Claudin 5
43	-2.3	*PTGDS*	Prostaglandin D2 synthase
44	-2.3	*RRAD*	Ras related glycolysis inhibitor and calcium channel regulator
45	-2.4	*ACKR1*	Atypical chemokine receptor 1 (Duffy blood group)
46	-2.5	*RSPO1*	R-spondin 1
47	-2.9	*RIMS2*	Regulating synaptic membrane exocytosis 2
48	-3.6	*TOR4A*	Torsin family 4 member A
49	-4.0	*ITIH1*	Inter-alpha-trypsin inhibitor heavy chain 1

Applying a threshold of fold change (log_2FC) 2 and adjusted *P*-value < 0.05, 49 differentially expressed genes contain a p52:RelB binding site +/-1kb from the transcription start site. Positive FC indicates expression is higher in CCS tumour CD45- cells relative to CD45- cells from normal skin.

Supplementary table 3. Top 50 differentially expressed genes in CCS scRNAseq clusters

CCS keratinocytes	Pericytes	Vascular endothelial cells	Fibroblasts
KRT23	RGS5	ECSCR	CFD
COL9A2	GJA4	ADGRL4	APOD
TRIM29	NDUFA4L2	CD93	DCN
BCL2A1	PROCR	SOX17	TNFAIP6
SAA1	KCNE4	FLT1	MMP2
CLDN4	EDNRB	PLVAP	LUM
FABP7	PLIN2	BCL6B	THBS2
KRT15	EDNRA	CLDN5	MEDAG
MIA	SLC25A4	PECAM1	CXCL12
KRT14	GPR4	TM4SF18	CXCL14
KRT5	ADIRF	EGFL7	CFH
CD70	TFPI	ZNF385D	PID1
NPPC	ISG15	STC1	ABI3BP
IRF6	LIF	STC2	PTGES
WFDC2	HES4	EMCN	CCDC80
LAMB3	CPE	MGST2	SRPX
CD24	CRISPLD2	CALCRL	CLMP
DSC2	EPAS1	SLCO4A1	CYP1B1
DSC3	DYNLT3	CDH13	SERPINE2
SPINT2	SRGN	IFI27	MT1M
SERPINB5	CXCL8	MMP1	TIMP1
TACSTD2	ADAMTS4	ANGPT2	PTGS2
KRT7	CREM	TRIB3	ALAS1
SFN	COL6A2	ENG	S100A4
KRT17	CXCL2	HLA-DRB1	MEG3
RBP1	CYCS	GNG11	IL6
ACTG2	GPRC5A	PRSS23	NR4A2
LAMC2	PHLDA1	AK1	LRP1
PERP	LAMA4	HLA-DPA1	NNMT
SH3RF1	PRKAR1A	ESAM	FGF7
SYNPO2	BNIP3L	HSD17B11	UAP1
DSP	HILPDA	PARVB	CXCL1
NEDD4L	IFITM1	COL15A1	PRRX1
KRT8	CXCL3	PGF	TNFAIP2
S100A2	PLPP3	FXYD5	CTSL
FHL2	ANKRD37	BACE2	GSN
CXCL16	PAM	SYNPO	ID3
FBXO32	LRRFIP1	PNP	SOD3
HIST1H2AC	SLC2A3	HLA-DQB1	IGFBP4
SDC4	CTSL	ST6GALNAC4	MGP

SFRP1	RASD1	TGFBR2	A4GALT
TNF	IFITM2	TCF4	VASN
SCPEP1	BGN	ARPC1B	MT1A
PAPSS1	VEGFA	EIF4EBP1	SERPING1
HIST1H1C	GBE1	FAM213A	UGDH
EGR1	CCDC107	ZEB1	ZEB2
DST	SOD3	SERPINE1	PGD
H1F0	SNHG15	PLAT	ZC3H12A
MARCKSL1	OPTN	SPRY1	MT2A

Supplementary figures

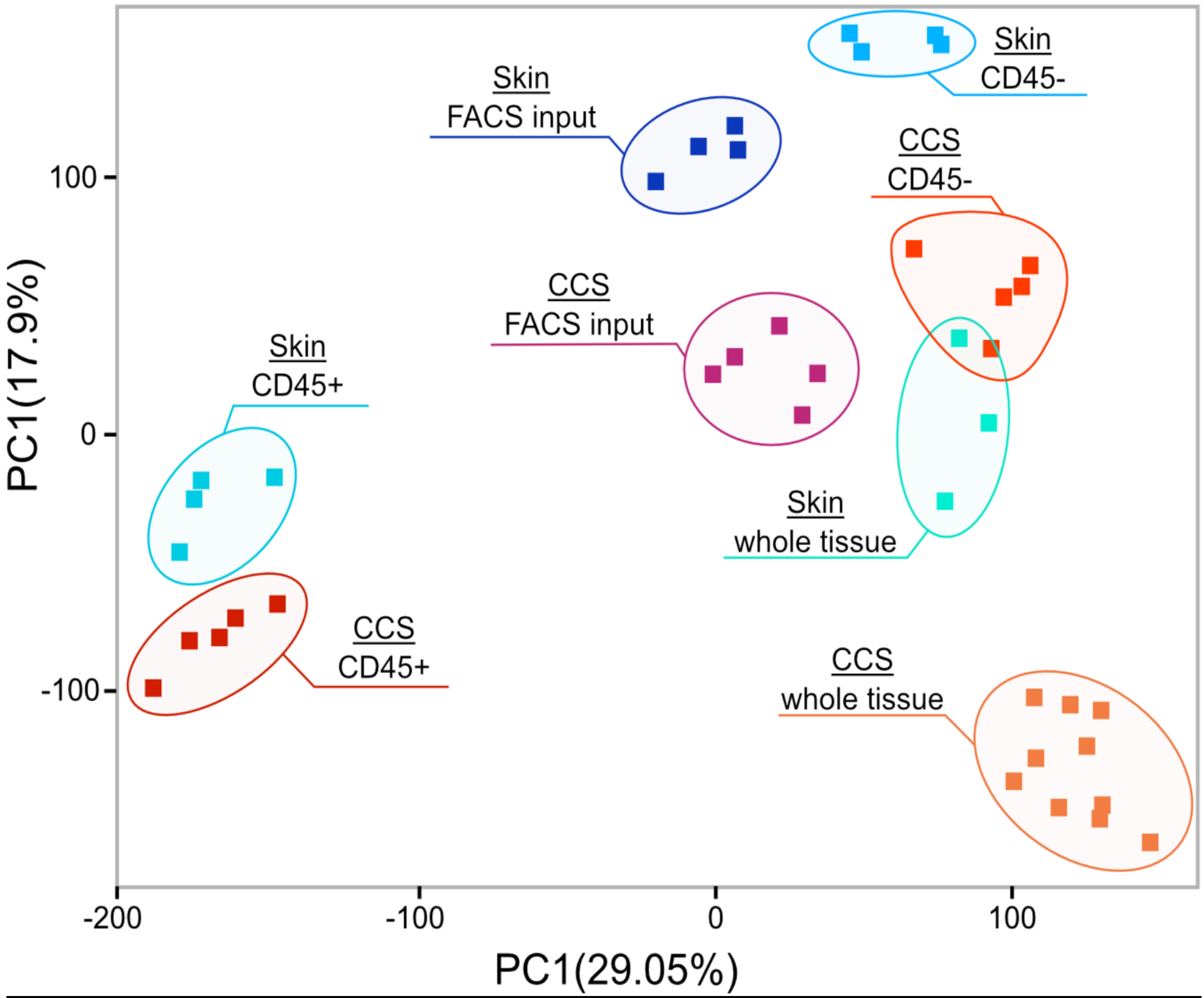

Supplementary Figure 1. Bulk RNA sequencing of CCS tumours and CD45+ and CD45- cell fractions. Principal component analysis of transcriptomic data from FACS sorted CCS tumours reveals distinct clusters of CD45- CCS and CD45- normal cells.

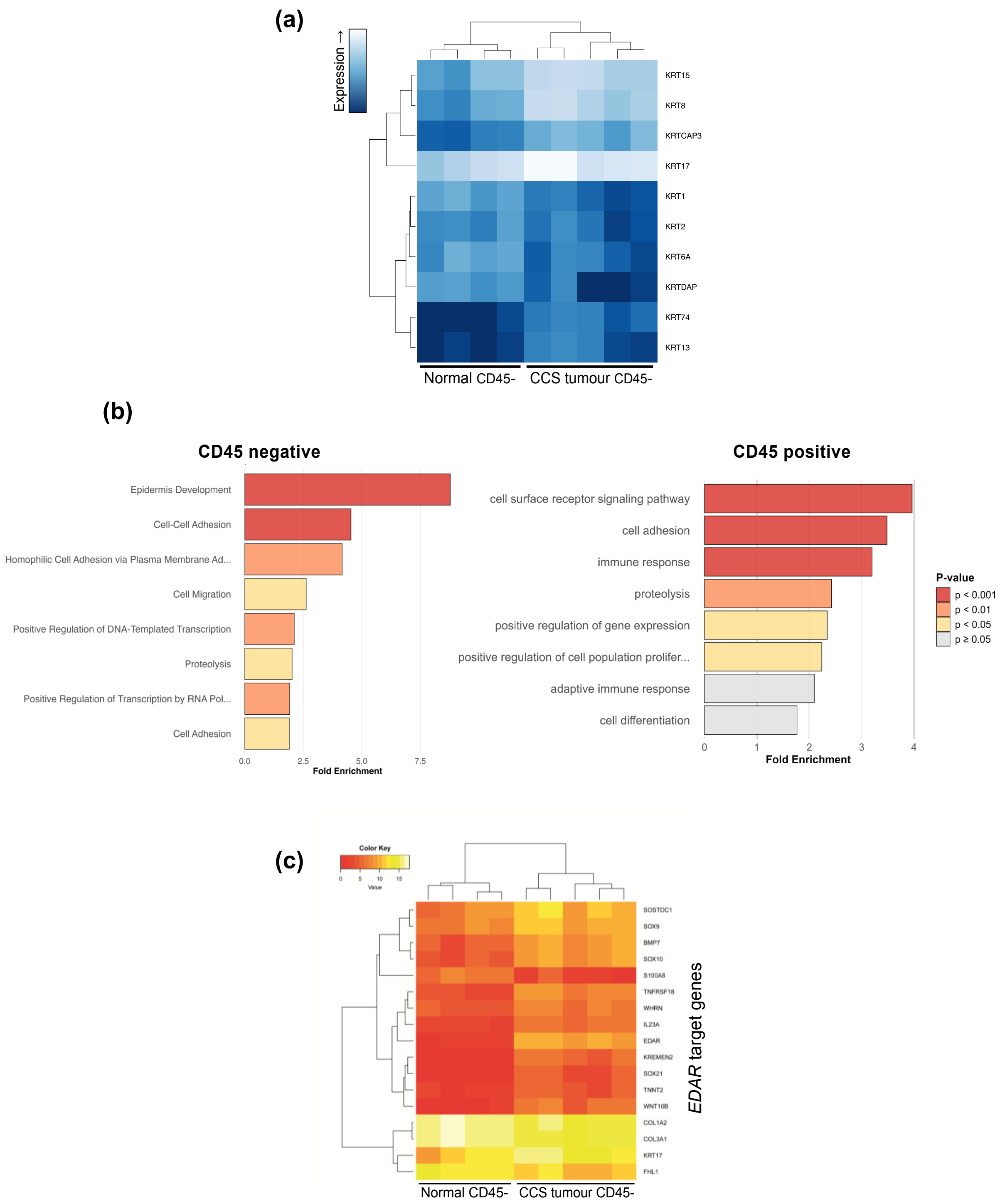

Supplementary Figure 2. Bulk RNAseq of CD45- CCS tumour cells demonstrate a distinct cytokeratin profile from CD45- control skin cells. A) CD45- cells isolated from CCS tumours and normal skin by FACS differentially express 10 protein coding keratin transcripts (log2FC>2, adjusted P-value <0.05). Dark blue indicates low expression, light blue indicates high expression. The gene encoding keratin 74 (KRT74), a hair follicle specific keratin, was ranked first by positive fold-change. **B)** Enriched biological processes in CD45$^{+/-}$ CYLD cutaneous syndrome tumour cells compared to CD45$^{+/-}$ healthy skin cells. **C)** CD45^{-} cells isolated from CCS tumours and normal skin by FACS differentially express 16 potential EDA/EDAR signalling pathway target genes (FC2, adjusted P-value < 0.05). Heatmap shows hierarchical clustering by Euclidean distance. Red indicates low expression, light-yellow indicates high expression.

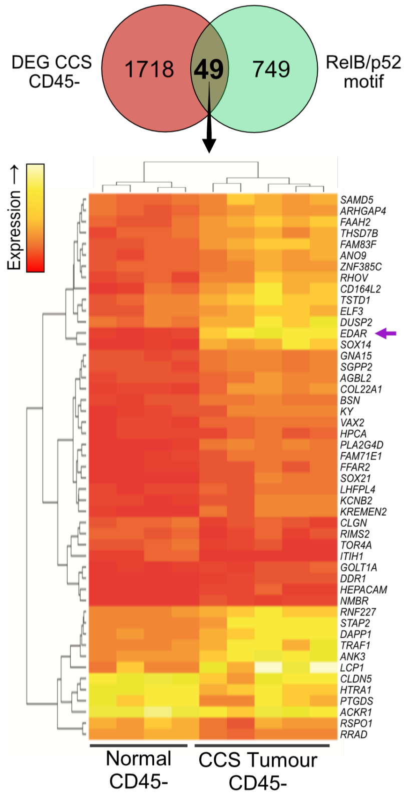

Supplemental Figure 3. An EDAR cluster is upregulated in bulk RNAseq data from flow sorted CD45- CCS tumour cells. 49 genes are differentially expressed between differentially expressed between FACS sorted CD45- CCS tumour and CD45- normal skin cells containing the p52/RelB motif GGGGNTTTCC. Hierarchical clustering by Euclidean distance of gene expression produces clusters within a heatmap. Red indicates low expression, light-yellow indicates high expression. EDAR had the greatest positive fold-change from 1718 DEGs (FC>2, adjusted P-value < 0.05) (purple arrow).

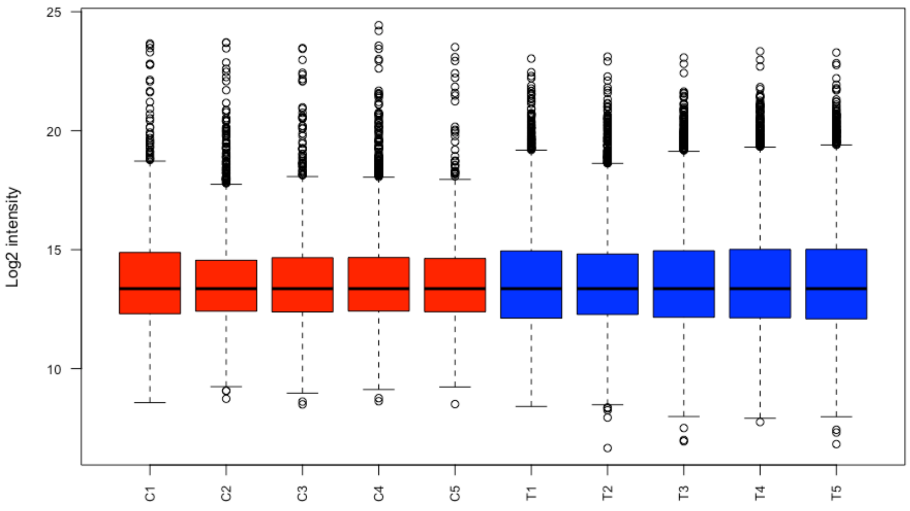

Supplementary Figure 4. Normalised log2 intensities of proteins detected in CD45- epidermis and CD45-/CD200- CCS tumour cells utilising mass spectrometry. Normalised log2 intensities of CD45- epidermis (C1-C5) and CD45-/CD200+ CCS tumour cells (T1-T5).

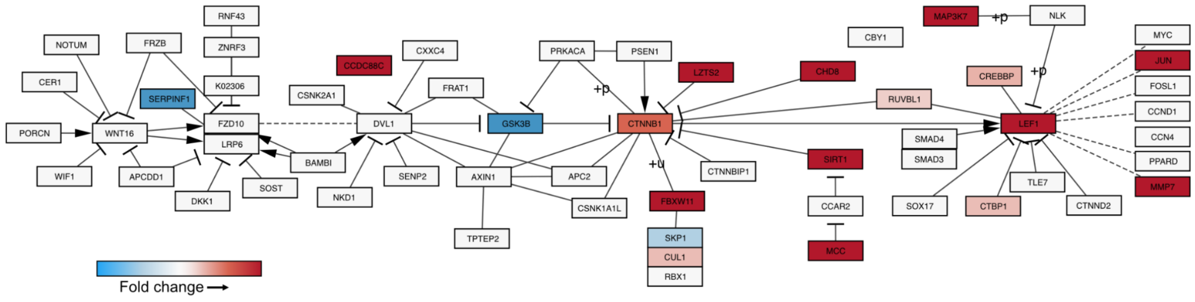

Supplementary Figure 5. **Wnt-Beta catenin signalling with differential protein expression between CCS CD45-CD200+ and normal skin CD45- cells detected using mass spectrometry and overlaid on a KEGG pathway.** Fold change is indicated as increasing (red) or decreasing (blue). Non-coloured proteins were not detected.

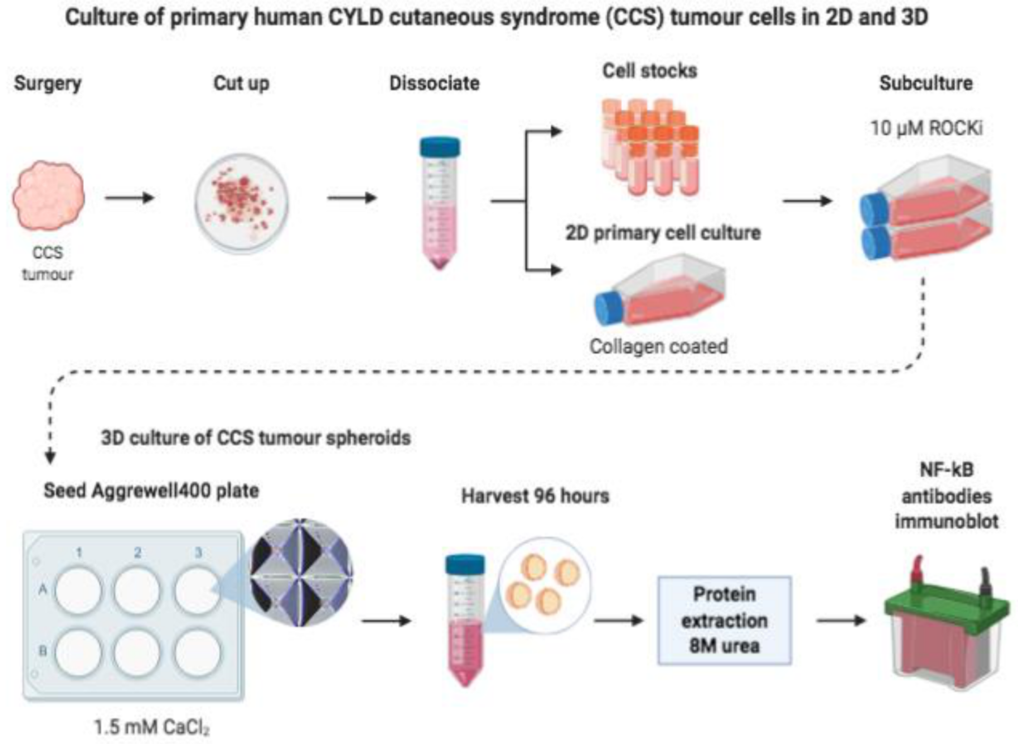

Supplementary Figure 6. **3D culture of primary human keratinocytes derived from CCS tumours.** Fresh CCS tumours were dissociated in 2.5% trypsin and 1 mg/ml collagenase at 37°C. 2D cultured cells were seeded on collagen coated T75 flasks and subcultured in keratinocyte-serum free media (KSFM) supplemented with 10 µM Y-27632 (ROCKi). Two confluent T75 flasks were pooled to seed an Aggrewell400 plate containing 7000 microwells per well. Within 24 h, one 3D spheroid aggregate formed per microwell. Spheroids were harvested at 96 h and protein extracted by sonication in 8M urea lysis buffer for immunoblot analysis of NF-κB signalling.

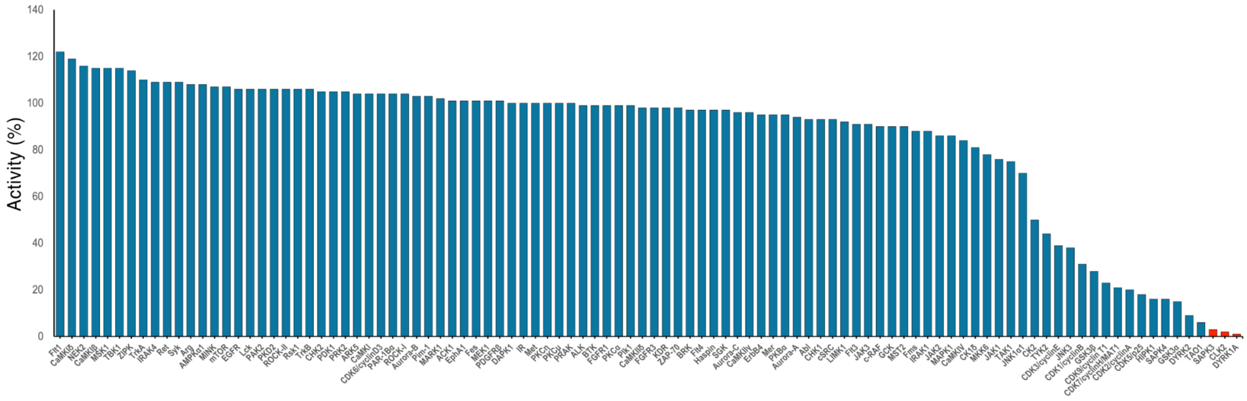

Supplementary Figure 7. Kinome profile for SU1664: % residual activity @ 1 µM (at ATP K_m).

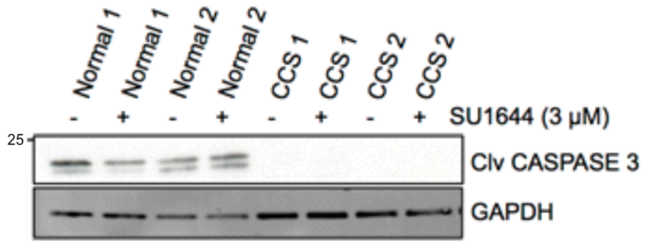

Supplementary Figure 8. Effects of SU1644 IKKα inhibition in 3D primary spheroids. Cleaved caspase 3 levels indicate that the normal primary spheroids were undergoing low levels of apoptosis in the culture model, which SU1644 (3uM) did not increase. CCS spheroids did not demonstrate any change in expression of cleaved caspase 3 following addition of SU1644 (3uM).

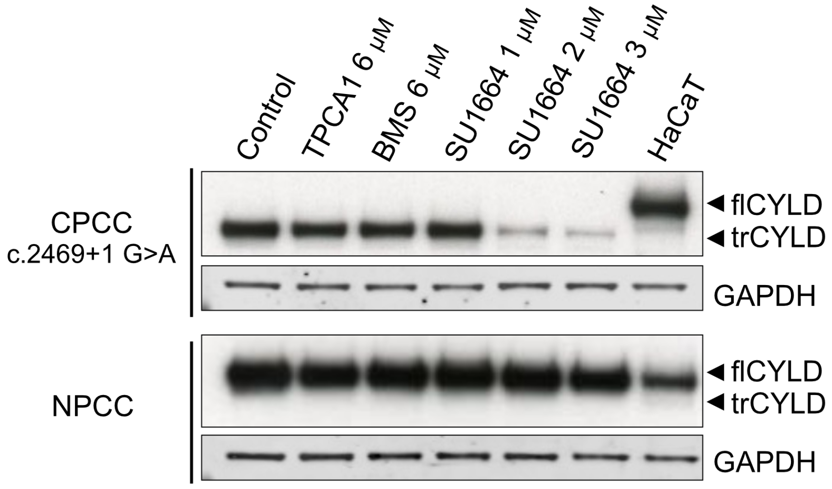

Supplementary Figure 9. Truncated CYLD levels are reduced in a splice site pathogenic variant of CYLD after SU1644 treatment. Levels of trCYLD (truncated CYLD) and flCYLD (full length CYLD) in CCS primary cell culture (CPCC) and normal primary cell culture (NPCC) detected by immunoblotting after 24 h treatment with IKK inhibitors. HaCaT lysate is included as a positive control for flCYLD. BMS; BMS345541.
